# Estimating the prevalence of *Echinococcus* spp. in a Tibetan fox (*Vulpes ferrilata*) population on the eastern Tibetan Plateau

**DOI:** 10.1186/s13071-025-07085-3

**Published:** 2026-01-21

**Authors:** Qingqiu Zuo, Xu Wang, Jiaxin Zheng, Gang Wang, Xiaodong Weng, Weibin Jiang, Nan Liu, Yingyuan Yang, Jue Chen, Zhuoma Bianba, Youzhong Ding, Xiaoming Wang, Xu Wei, Zhenghuan Wang

**Affiliations:** 1 National Institute of Parasitic Diseases, Chinese Center for Disease Control and Prevention (Chinese Center for Tropical Diseases Research), NHC Key Laboratory of Parasite and Vector Biology, National Key Laboratory of Intelligent Tracking and Forecasting for Infectious Diseases, WHO Collaborating Centre for Tropical Diseases, National Center for International Research On Tropical Diseases, Shanghai, China; 2 School of Life Sciences, East China Normal University, Shanghai, China; 3 National Health Commission Key Laboratory of Echinococcosis Prevention and Control, Xizang Center for Disease Control and Prevention, Lhasa City, Xizang Autonomous Region China; 4 Shanghai Science and Technology Museum, Shanghai, China

**Keywords:** *Echinococcus* spp., Tibetan fox, Copro-DNA genotype, Fecal prevalence, Population prevalence, Sampling technique

## Abstract

**Background:**

The Tibetan fox (*Vulpes ferrilata*) is a crucial wild definitive host of *Echinococcus* cestodes on the Tibetan Plateau. Fecal detection of *Echinococcus* DNA (fecal prevalence) is commonly used to estimate *Echinococcus* spp. prevalence in canine populations (population prevalence). However, this approach may be biased without individual identification, when the same individuals are repeatedly sampled, potentially leading to the misestimation of exact population prevalence.

**Methods:**

Fecal samples collected from Tibetan foxes in Shiqu County (2010–2012) were genotyped to identify individual foxes, followed by copro-PCR to determine the population prevalence of *Echinococcus* spp. in the genotyped foxes. A virtual resampling program was developed to assess sampling bias and determine the optimal interval between sampling line transects. The derived optimal interval was then applied in surveillance conducted in 2015, 2016, and 2019.

**Results:**

In total, 679 Tibetan fox feces were confirmed from 1219 field-collected samples (2010–2019). From 250 samples (2010–2012), 61 distinct fox individuals were identified. Virtual resampling analysis determined the optimal sampling interval to be 200–900 m, confirming fecal prevalence as an unbiased estimator of population prevalence. The implementation of a 500 m sampling interval in the surveillance of *Echinococcus* spp. (2010–2019) revealed an overall prevalence of 45.7% (95% CI 41.4–50.0%), with 32.3% (28.4–36.2%) for *Echinococcus multilocularis* and 23.5% (19.8–27.2%) for *Echinococcus shiquicus*. Mixed infections were detected annually, with an overall prevalence of 11.1% (8.4–13.8%). Significant temporal reductions were observed in the prevalence of *E. multilocularis* (*Z* = − 4.640, *P* < 0.001), mixed infections (*Z* = − 3.896, *P* < 0.001), and overall *Echinococcus* spp. (*Z* = − 2.155, *P* = 0.031). The prevalence trends of *E. multilocularis* and *E. shiquicus* were significantly associated, showing an inverse relationship (*χ*^2^ = 68.861, *P* < 0.001).

**Conclusions:**

A 200–900 m interval between feces sampling line transects was established as the optimal distance for assessing the prevalence of *Echinococcus* spp. in the Tibetan fox population. The persistent high prevalence of *Echinococcus* spp. in the Tibetan fox indicates an ongoing sylvatic transmission risk in Shiqu County. The opposing prevalence trends between *E. multilocularis* and *E. shiquicus* indicated a complex interaction within their shared host, warranting further study.

**Graphic Abstract:**

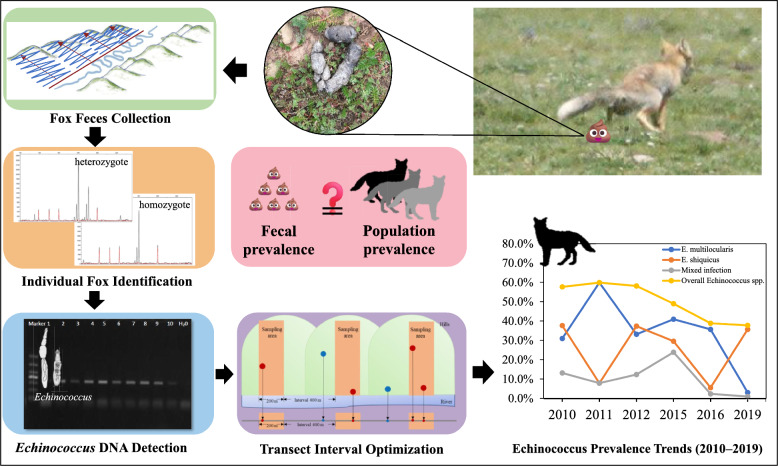

**Supplementary Information:**

The online version contains supplementary material available at 10.1186/s13071-025-07085-3.

## Background

Echinococcosis caused by *Echinococcus* species is a lethal zoonosis worldwide, especially in the northern hemisphere [1]. Transmission of this severe disease involves complex cycles between the definitive carnivorous hosts and the intermediate herbivorous hosts [2]. Human infection typically occurs through the accidental ingestion of *Echinococcus* eggs excreted by definitive hosts [2]. The parasite is disseminated in the environment through the spatial activities and excretory behaviors of the definitive hosts [3, 4]. Therefore, assessing the prevalence of *Echinococcus* spp. in its definitive host populations provides essential knowledge about the risks and significance of echinococcosis in local areas.

The eastern Tibetan Plateau has been identified as a hyperendemic area for echinococcosis, with the presence of three *Echinococcus* species coexisting in this region [5, 6]. *Echinococcus granulosus* (s.l.) primarily infects domestic animals and causes cystic echinococcosis in humans [1]. In contrast, *E. multilocularis* and *E. shiquicus* Xiao, Qiu, Nakao, Li, Yang, Chen, Schantz, Craig & Ito, 2005 [7, 8] share an overlapping transmission cycle involving multiple wildlife hosts, with the Tibetan fox serving as the definitive host and several species of small mammals (rodents and pikas) as intermediate hosts [8–10]. In contrast to *E. multilocularis*, the causative agent of alveolar echinococcosis, the most lethal form of echinococcosis, *E. shiquicus* has not yet been documented to infect humans. However, its co-infection with *E. multilocularis* in local domestic dog populations [11, 12] highlights the need for comprehensive monitoring of both *Echinococcus* species in the wildlife reservoir to facilitate a thorough assessment and warning system for the epidemiology of echinococcosis in the region. As a fundamental measure in monitoring zoonotic diseases, the periodic evaluation of the prevalence of *Echinococcus* spp. in the Tibetan fox population can provide essential and pivotal parasitological data for the epidemiology and prevention of echinococcosis in local areas.

Testing molecular prevalence based on the noninvasive feces sampling technique and copro-DNA analysis (hereafter ‘fecal prevalence’) is a practical and humane approach to evaluating the parasite prevalence in living host populations (hereafter ‘population prevalence’), especially for wild Carnivora [13–15]. The Tibetan fox is an endangered wildlife species under national second-class protection in China [16], and fecal prevalence has been widely evaluated in various studies [17–19]. Fecal samples are usually collected with line transect surveys in the field. The Tibetan fox is a territorial species with an average territory size of 286.0 ± 137.6 ha [20], which it strongly protects and from which it largely excludes other foxes [20, 21]. If line transects are set too densely, fecal samples from the same individual fox may be sampled multiple times. Fecal prevalence would thus be significantly biased relative to the actual population prevalence due to the repeated testing of a small group of individuals. On the contrary, if the line transects are too sparse, infection information will be lost with the loss of fecal samples, resulting in the misestimation of parasite infection.

To resolve this problem, both the molecular identification of *E. multilocularis* and the simple sequence repeat (SSR) technique of individual genotyping were applied to test copro-DNA materials when evaluating the prevalence of *E. multilocularis* in a coyote population in Canada [22]. Individual genotyping techniques using multiple SSR loci in copro-DNA have also been developed for the Tibetan fox in our previous studies [23, 24], providing a way to evaluate the prevalence of *Echinococcus* spp. in a Tibetan fox population based on the recognition of individual hosts. However, the disadvantages of this protocol, including the high cost and low efficiency of the SSR technique, have been pointed out [23]. The tedious analysis procedure prevents the generation of rapid real-time prevalence data, especially in large-scale annual surveys.

In this study, a revised field sampling technique for Tibetan fox feces was developed, using an optimal spatial interval between line transects to control for repetitive sampling, as an efficient and practical method to control potential deviation in fecal prevalence from the actual population prevalence. By integrating the copro-DNA genotyping technique and a copro-PCR diagnostic analysis, we initially determined the individual identity and the *Echinococcus* spp. infection status of each fecal sample originating from Tibetan foxes in Shiqu County in 2010–2012. We then calculated the molecular population prevalence of *Echinococcus* spp. in the Tibetan fox population based on the individual foxes identified. A virtual feces resampling program was then used to determine the optimal size of the regular intervals between line transects required for fecal prevalence to provide an unbiased estimate of the population prevalence in the perceived fox population each year. Finally, a fecal sampling protocol with this optimal line transect interval was used to evaluate the molecular prevalence of *Echinococcus* spp. in the Tibetan fox population in Shiqu County in 2015, 2016, and 2019.

## Materials

### Study area

Field studies were performed in an area of *c*. 60 km^2^ in Yongbo Valley (32°19′–34°20′N, 97°20′–99°15′E), Shiqu County, Ganzi Tibetan Autonomous Prefecture, Sichuan Province, China, with an average elevation > 4300 m above sea level, where the highest human infection rates of *E. multilocularis* (alveolar echinococcosis) in the world have been recorded [5, 6]. A typical grassland area on the eastern Tibetan Plateau, the study area comprises gently rolling hills and broad valleys, with rivers passing through the bottoms. The landcover is primarily *Kobresia* meadow, whereas shrubs, mainly *Potentilla fruticosa* L. and *Salix cupularis* Rehder, occur from the middle to the tops of some hill slopes.

### Fecal samples collection in 2010–2012

Fox feces were mainly identified by their characteristics (e.g., color, shape, moisture, and smell) [10, 17]. Briefly, during July and August 2010–2012, a sweeping sampling technique with zigzag paths was used to cover the entire study area. Extra attention was paid to potential dens and scent marking points (stones, dirt mounds, bones of large animals). All fox feces detected were collected, and each specimen was stored separately in a 50 mL centrifuge tube with 75% ethanol and kept for at least 3 weeks at − 80 °C for safety before further processing. The global positioning system (GPS) coordinates of the location of each fecal sample were obtained with a handheld GPS receiver (Garmin GPS 72H, Garmin Ltd., Olathe, KS, USA) and recorded for further spatial analysis of the hosts.

### DNA extraction

About 4 g of each fecal sample was dissolved in 50 mL of deionized H_2_O, emulsified at 80 °C for 10 min, and then filtered with two layers of sterilized medical gauze to remove large particles. The filtrate containing host cells, taeniid tissues, and eggs was centrifuged at 2320 × g for 30 min. The sediment was then re-emulsified in 600 μL of Buffer ASL (QIAamp DNA Stool Mini Kit, Qiagen, Hilden, Germany) and divided into sample A and sample B. Sample A was used to extract genomic DNA for host species identification, sex determination, and individual genotyping. Sample B was used to extract the DNA from parasite eggs for the detection of *Echinococcus* species. This included a mechanical disruption procedure to break the eggshells of *Echinococcus* spp., whereas sample A was not subjected to this mechanical disruption to preserve the integrity of host DNA for SSR genotyping. Briefly, sample B was added to a Precellys^®^ tube (2 mL, Peqlab Biotechnology, Erlangen, Germany) with 200 mg of Precellys^®^ ceramic beads (0.5 mm, Peqlab Biotechnology, Erlangen, Germany) and subjected to two rounds of homogenization at 5500 rpm for 15 s in a Bertin Precellys^®^ 24 homogenizer (Bertin Technologies, Aix en Provence, France). DNA was extracted from both samples A and B with the QIAamp DNA Stool Mini kit (Qiagen, Hilden, Germany), according to the manufacturer’s instructions. The protocol included the use of InhibitEX Buffer (QIAamp DNA Stool Mini Kit, Qiagen, Hilden, Germany) to control the impact of PCR inhibitors.

### Fox species and sex identification

Because the rare red fox (*Vulpes vulpes* (Linnaeus, 1758)) has a sympatric distribution in this area, a PCR-restriction fragment length polymorphism (RFLP) assay was used to check the species origin of the fecal samples [25]. Briefly, to the DNA extracted from each sample A, a *c.* 478-bp fragment of the mitochondrial cytochrome *b* gene (*CYTB*) was amplified and digested separately with the *Bam*HI and *Ssp*I restriction enzymes. Tibetan fox DNA was digested with BamHI, generating three bands of approximately 220 bp, 140 bp, and 120 bp, whereas red fox DNA was digested with *Ssp*I, producing three bands of approximately 270 bp, 120 bp, and 90 bp.

The successfully identified Tibetan fox fecal samples were further evaluated to determine the sex. A 105-bp fragment of the sex-determining region Y (*SRY*) gene was amplified, using a 195-bp fragment of the *X*-linked zinc finger protein (*ZFX*) gene as the internal control. We deemed a fox to be female when only the 195 bp fragment was generated, and to be male when both the 195 bp and the 105 bp fragments were generated. The reaction system and amplification conditions for the PCR were as described previously [10]. Samples that failed to meet these criteria were defined as ‘sex unknown’.

### SSR genotyping

In our previous studies [23, 24], a total of 21 SSR markers (Additional file l: Table S1) were tested in the genotyping of fecal samples from the Tibetan fox. Each SSR locus was amplified with PCR in a total reaction volume of 15 μL containing 2.5 μL of copro-DNA from sample A, 7.5 μL of Premix Ex Taq (1.25 U/25 μL; TaKaRa, Biotechnology, Dalian, China), 0.6 μL of each primer, 0.6 μL of bovine serum albumin (BSA; 20 mg/mL; TaKaRa, Biotechnology, Dalian, China) to mitigate potential PCR inhibition, and 3.2 μL of double-distilled water (ddH_2_O; TaKaRa, Biotechnology, Dalian, China). Amplification was performed on the Bio-Rad DNA Engine PTC-200 (Bio-Rad, Hercules, CA, USA) under the following conditions: 95 °C for 5 min; 35 cycles of 95 °C for 30 s, 54–60 °C for 30 s, 72 °C for 45 s; and then 72 °C for 10 min. All positive PCR products, detected with 2% agarose gel electrophoresis, were genotyped by fragment analysis using an ABI 3730 XL DNA Analyzer (service provided by Sangon Biotech, Co., Ltd (Shanghai, China). Alleles were then scored based on size using GeneMapper 4.0 (Applied Biosystems, Beijing, China).

To reduce any errors in genotyping, at least four independent amplifications for each SSR locus were performed. In the consensus definition of genotypes, a genotype was deemed ‘homozygous’ when four replicates yielded only one allele at the same locus, but ‘heterozygous’ when two alleles were detected at the same locus. When more than two alleles were detected at the same locus when four replicates of a fecal sample were analyzed, another two amplifications were performed, and the two alleles detected most frequently in the six replicates were deemed to be the genotype of that heterozygous locus. After six rounds of amplification, SSR loci that failed to conform to these criteria were excluded.

After amplification, the success rate of amplification, the number of alleles (N), the polymorphism information content (PIC), the expected heterozygosity (He), and the observed heterozygosity (Ho) of the samples were calculated with Cervus version 3.0.7 [26]. The probability of identity (P_ID_) of the loci combination was calculated as P_ID*biased*_ (for randomly mating individuals), P_ID*unbiased*_ (corrected for sample size), and P_ID*sibs*_ (for related individuals) with Gimlet 1.3.1 [27].

The quality of copro-DNA can be significantly influenced by complex field factors and the diets of the host individual [25]. Therefore, the amplification of our 21 SSR markers performed quite differently with fecal samples collected in different years. Based on the characteristics of each locus (e.g., amplification success, number of alleles, polymorphism information), we finally selected nine SSR loci without significant linkage disequilibrium that showed stable amplification from the fecal samples (see Additional file 1: Fig. S1, Table S1). In detail, six of the nine loci (P01i, P02d, P08, P05h, CXX172, and AHT-142) showed the best performance and sufficient power (i.e., probability of identity, P_ID_) to distinguish the identities of the fecal samples collected in 2010, whereas another combination of six loci (Cph6, P08, Cph8, P03, CXX172, and P01i) showed the best performance in genotyping the fecal samples collected in 2011 and 2012.

### Discrimination of individual Tibetan foxes

To identify individual foxes between 2010 and 2012, we utilized a year-specific optimal set of six SSR loci (markers) in each year, and the sex identification result (as the seventh marker), as described in the preceding sections (Additional file 1: Fig. S1). The criteria for individual identification were as follows: (i) A sample was used for individual identification if at least six of its corresponding seven markers were successfully amplified. (ii) Considering the low amplification success of copro-DNA, fecal samples with genotypes differing at no more than two alleles and without a sex mismatch were judged to be from the same individual fox. (iii) The sex of those identified fox individuals with missing sex identification information or ambiguous *SRY* amplification was considered ‘unknown’.

### *Echinococcus* species detection

For those feces confirmed to be of Tibetan fox origin, the copro-DNA of sample B from each fecal sample was used to screen for *Echinococcus* species. To detect the presence of *Echinococcus granulosus* (Batsch, 1786), an external primer pair was used to amplify a 269-bp fragment of repeated sequence (EgG1 *Hae* III) in the genome, and then an internal primer was used to amplify a 133-bp segment inside this fragment [28]. To identify *E. multilocularis* and *E. shiquicus*, an 874-bp fragment of the *cox1* gene was amplified with a universal primer pair for the family Taeniidae [29]. Two other species-specific primer pairs were then used to amplify an internal fragment to identify *E. multilocularis* [30] and *E. shiquicus* [31]. Details of the primers are listed in Additional file l: Table S1. All PCRs were performed in a 20 μL reaction containing 2 μL of extracted DNA, 0.8 μL of each primer, 1 μL of BSA, 10 μL of Premix Ex Taq, and 5.4 μL of ddH_2_O. The thermal cycling parameters were 35 cycles of 94 °C for 30 s, 52–55 °C (Additional file 1: Table S1) for 45 s, 72 °C for 90 s, and then 72 °C for 10 min, performed on the Bio-Rad DNA Engine PTC-200. Positive PCR products confirmed with 2% agarose gel electrophoresis were sequenced by Sangon Biotech and compared with the NCBI database (http://www.ncbi.nlm.nih.gov/BLAST) for *Echinococcus* species identification.

### Molecular estimation of *Echinococcus* spp. population prevalence

The molecular prevalence of *Echinococcus* spp. in each year from 2010 to 2012 was evaluated from fecal samples of Tibetan fox origin (fecal prevalence) and the fox individuals identified (population prevalence) in that year. The fecal prevalence of *Echinococcus* spp. was calculated as the occurrence of positive fecal samples in percentage. Population prevalence was calculated as the occurrence (in percentage) of individual Tibetan foxes (identified with the SSR technique) whose fecal samples were positive for *Echinococcus* spp.

### Estimation of the optimal range of intervals between fecal sampling line transects

To ensure that fecal prevalence is an effective estimate of population prevalence, considering the expenditure, workforce, and efficiency required, a confirmed optimal regular interval between line transects is essential. The distance between perceived fox identities (the geometric center of fecal samples assigned to the same individual) was calculated to restrict the appropriate interval range for a standard line transect in the feces sampling protocol. Line transects with regular intervals between them were then designed on a digital map to cover the entire study area, using ArcMap 10.2 (ESRI, 2013). Each line transect started at the edge of the river at the bottom of the valley and proceeded vertically to the ridge of the valley. A series of sampling areas, 100 m wide, on each side of the line transects (i.e., 200 m wide, in total) were inspected. All Tibetan fox feces detected in these sampling areas were collected to evaluate the fecal prevalence of *Echinococcus* spp., as described above.

**Fig. 1:**
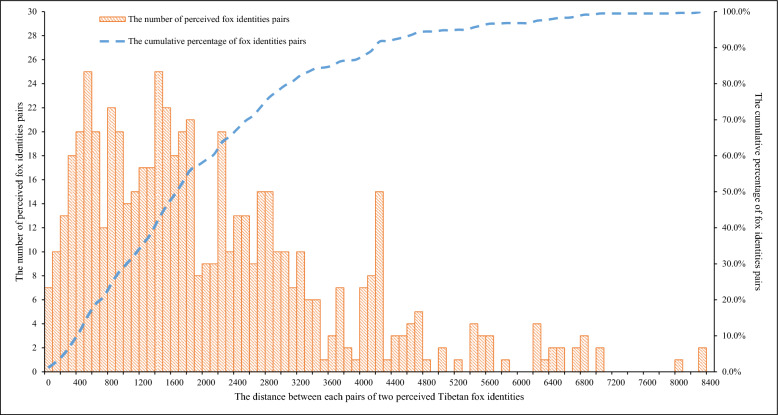
Distances between each two fox identities

We first marked the GPS locations of all Tibetan fox feces collected from 2010 to 2012 on a digital map of the study area. Virtual line transects at regular intervals were then designed on the map based on the feces locations for each year. According to the spatial distances between feces from different foxes (see the Results, Fig. 1), 29 regular intervals, from 200 to 3000 m, at increments of 100 m, were tested. To each interval, a series of virtual line transects was arranged one by one with this fixed interval to cover the entire study area by setting a random starting point for the first virtual line transect at the bank of the river, through the bottom of the valley. Feces located in the area within 100 m of each virtual line transect were resampled to calculate the fecal prevalence of *Echinococcus* spp. This virtual resampling process and calculation of fecal prevalence were repeated 30 times for each regular interval. The deviation of fecal prevalence from population prevalence was evaluated with a *t-*test [32]. Fecal prevalence was considered an effective estimate of population prevalence when the results for all four types of fecal prevalence (*E. multilocularis*, *E. shiquicus*, mixed infections, and overall *Echinococcus* spp.) did not deviate significantly from the population prevalence. Conversely, if any result for the four types of fecal prevalence deviated significantly from the population prevalence, then fecal prevalence was considered an ineffective estimate. The number of effective estimates in the test for each interval was recorded. When the fecal prevalence results of all 30 resampling processes did not deviate significantly from the population prevalence, this range of intervals was judged to be the optimal range of regular intervals. The resampling protocol (Additional file 1: Fig. S2) and *t-*test were all programmed with R 3.4.0 (http://www.r-project.org).

### Fecal sample collection and processing in 2015, 2016, and 2019

Among the range of optimal intervals between line transects determined in this study (refer to the section above), the optimal interval (i.e., 500 m) between line transects was selected for collecting fecal samples in July and August in 2015, 2016, and 2019, based on fiscal capacities and personnel availability (refer to the optimal range of intervals between line transects given in the Results). Each line transect commenced at the edge of the river at the bottom of the valley and proceeded vertically to the ridge of the valley. A sampling area of 100 m wide on each side of the line transect (i.e., a total width of 200 m) was surveyed. Feces detected in the sampling area were collected, and the molecular prevalence of *Echinococcus* spp. was estimated as described above.

### Statistics

The *χ*^2^ goodness-of-fit test was used to evaluate the bias in the sex ratio of the Tibetan fox population. To compare the prevalence of *Echinococcus* spp. among different years, we calculated the fecal prevalence with the optimal sampling interval (i.e., 500 m) of infections for each of the three *Echinococcus* species, mixed infections of at least two species detected in the same fecal sample, and total infections of all *Echinococcus* spp. detected in each of the 6 years (2010, 2011, 2012, 2015, 2016, 2019) from 2010 to 2019. We then calculated the trend in *Echinococcus* spp. prevalence over time with the Cochran–Armitage test [33]. Differences in the prevalence of *Echinococcus* species were evaluated with the *χ*^2^ test. All statistical analyses were performed in R 3.4.0.

## Results

### Estimated Tibetan fox population from 2010 to 2012

A total of 1219 fecal samples were collected from 2010 to 2019, and 55.7% (680/1219) of them were identified as originating from Tibetan foxes (Table 1). All 426 samples collected from 2010 to 2012 were used to estimate the fox population. Copro-DNA was successfully extracted from 62.7% (267 of 426) of the fecal samples, and 93.6% (250/267) of the extracted copro-DNA (confirmed to originate from Tibetan foxes) was used for SSR genotyping. Of these fecal samples, 37.6% (94 of 250) were qualified and retained for the discrimination of individual foxes.

**Table 1:** Information on fecal samples from 2010 to 2019

	2010	2011	2012	2015	2016	2019	Total
Fecal samples	187	146	93	326	367	100	1219
Tibetan fox feces	121 (52♂:42♀)	69 (28♂:23♀)	60 (19♂:28♀)	175	159	95	679
Genotyping-qualified	28 (23.1%)^a^	38 (55.1%)	28 (46.7%)	–	–	–	94 (37.6%)
Genotyped individuals	18 (6♂:5♀)	24 (9♂:8♀)	19 (8♂:5♀)	–	–	–	61 (23♂:18♀)

^a^Proportion of feces qualified for genotyping and retained from the total samples

In the discrimination of individual Tibetan foxes, when we considered all six SSR loci and sex markers (*ZFX*/*SRY*), the probability of identity (P_ID_) was always < 0.01 in distinguishing fecal samples, regardless of whether they were calculated with or without correction for sample size (P_ID*unbiased*_ and P_ID*biased*_) or related individuals (P_ID*sibs*_, Additional file 1: Fig. S1). When we genotyped them with this combination of seven genetic markers, we identified 94 Tibetan fox fecal samples as deriving from 61 individual foxes (Table 1). Based on all 192 fecal samples and the 41 individual foxes that were successfully sexed (Table 1), the sex ratio of the fecal samples did not deviate significantly from 1:1 (1.06, 99♂: 93♀, *χ*^2^ = 0.188, *P* = 0.665). Similarly, the sex ratio of the Tibetan fox population based on the individual foxes identified did not deviate significantly from 1:1 (1.28, 23♂: 18♀, *χ*^2^ = 0.610, *P* = 0.435).

### Optimal range of intervals between line transects

From all 61 perceived fox individuals identified between 2010 and 2012, a dataset of 600 distances between each two identities detected in the same year was obtained. The average distance was 2097.9 ± 1549.4 m (19.2 m to 8348.8 m). Notably, approximately 49.3% of these distances fell within the range of 400–2000 m, whereas approximately 77.8% were < 3000 m (Fig. 1).

**Fig. 2:**
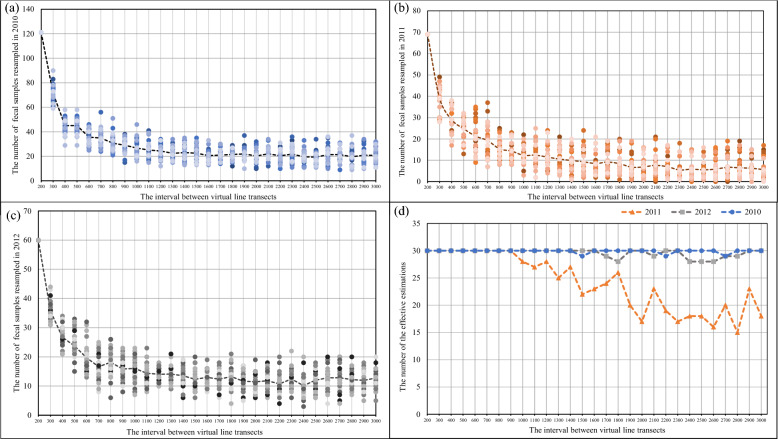
Number of resampled fox fecal samples in each test of each interval in 2010 (**a**), 2011 (**b**), and 2012 (**c**). Number of effective estimates for each interval (**d**). Related data and statistical results are presented in Tables S4–S6

Consequently, the intervals between virtual line transects were set from 200 to 3000 m, in increments of 100 m, for testing. Therefore, for fecal samples collected yearly from 2010 to 2012, 29 regular intervals were tested in each year. For data for all 3 years, the number of fecal samples resampled decreased when the regular interval between the virtual line transects increased from 200 to 1500 m (Fig. 2a–c, Table S2). Ineffective estimation of the population prevalence of *Echinococcus* species in 2011 was first detected when the interval was increased to 1000 m (Fig. 2b, Table S3). In the data for 2010 and 2012, ineffective estimation was detected when the interval was increased to 1500 m and 1700 m, respectively (Fig. 2a, c, Table S3). Therefore, based on the fecal samples collected from 2010 to 2012, when the regular intervals between the virtual line transects were between 200 and 900 m, resampled fecal prevalence was an effective estimate of all combinations of *Echinococcus* prevalence in the perceived Tibetan fox population (Fig. 2d, Table S3). To ensure the efficiency of fecal prevalence in the face of logistic constraints, we used 500 m as the optimal interval for sampling Tibetan fox feces with field line transects in subsequent years.

### Molecular prevalence of *Echinococcus* spp. in the Tibetan fox population in 2010–2019

When the data for all the qualified fecal samples from 2010 to 2019 were combined, the overall molecular prevalence of *Echinococcus* spp. was 45.7% (95% confidence interval [CI]: 41.4–50.0%), whereas the prevalences of *E. multilocularis* and *E. shiquicus* were 32.3% (28.4–36.2%) and 23.5% (19.8–27.2%) respectively. The prevalence of mixed infections of *E*. *multilocularis* and *E. shiquicus* was 11.1% (8.4–13.8%) (Table 2). No *E*. *granulosus* DNA was detected in the fecal samples during the study period (Table 2). The prevalence trends for *E. multilocularis*, mixed infections, and overall *Echinococcus* spp. from 2010 to 2019 declined significantly with time, whereas the prevalence of *E. shiquicus* showed no significant change (Table 2). A significant negative correlation was found between the prevalence of *E. multilocularis* and *E. shiquicus* (*χ*^2^ = 68.861, *P* < 0.001), where the increasing prevalence of one species was generally concurrent with the decreasing prevalence of the other.

**Table 2:** Prevalence of *Echinococcus* species (%, no. positive samples/total no. samples, 95% confidence intervals) and the trends in *Echinococcus* species prevalence from 2010 to 2019

	*E*.* multilocularis*		*E*.* shiquicus*		Mixed infection		Overall *Echinococcus* spp.	
2010^a^	31.1 (14/45, 17.6–44.6)	33.3 (6/18, 11.5–55.1)	37.8 (17/45, 23.6–52.0)	33.3 (6/18, 11.5–55.1)	13.3 (6/45, 3.4–23.2)	16.7 (3/18, 0–33.9)	57.8 (26/45, 43.4–72.2)	50.0 (9/18, 26.9–73.1)
2011^a^	60.0 (15/25, 40.8–79.2)	50.0 (12/24, 30.0–70.0)	8.0 (2/25, 0–18.6)	4.2 (1/24, 0–12.2)	8.0 (2/25, 0–18.6)	4.2 (1/24, 0–12.2)	60.0 (15/25, 40.8–79.2)	50.0 (12/24, 30–70)
2012^a^	33.3 (8/24, 14.4–52.2)	31.6 (6/19, 10.7–52.5)	37.5 (9/24, 18.1–56.9)	42.1 (8/19, 9.4–64.3)	12.5 (3/24, 0–25.7)	15.8 (3/19, 0–32.2)	58.3 (14/24, 38.6–78.0)	57.9 (11/19, 35.7–80.1)
2015	41.1 (72/175, 33.8–48.4)		29.7 (52/175, 22.9–36.5)		24.0 (42/175, 17.7–30.3)		49.1 (86/175, 41.7–56.5)	
2016	35.8 (57/159, 28.3–43.3)		5.7 (9/159, 2.4–9.0)		2.5 (4/159, 0.4–4.6)		39.0 (62/159, 31.4–46.6)	
2019	3.2 (3/95, 0.2–6.2)		35.8 (34/95, 26.7–44.9)		1.1 (1/95, 0–2.6)		37.9 (36/95, 28.1–47.7)	
Total	32.3 (169/523, 28.4–36.2)		23.5 (123/523, 19.8–27.2)		11.1 (58/523, 8.4–13.8)		45.7 (239/523, 41.4–50.0)	
Cochran–Armitage test for trends	*Z* = − 4.146↓	*Z* = − 4.640↓	*Z* = − 1.464	*Z* = − 0.196	*Z* = − 3.425↓	*Z* = − 3.896↓	*Z* = − 3.250↓	*Z* = − 2.155↓
	*P* < **0.001**	*P* < **0.001**	*P* = 0.143	*P* = 0.844	*P* = **0.001**	*P* < **0.001**	*P* = **0.001**	*P* = **0.031**

^a^Two prevalence estimates are provided for 2010–2012. Fecal prevalence (left subcolumn) was calculated from feces resampled at 500 m intervals, whereas population prevalence (right subcolumn) was derived from perceived fox individuals. Values in bold indicate statistically significant difference (*P* < 0.05).

## Discussion

In this study, by integrating parasitological diagnosis with a fecal genotyping analysis, we have demonstrated that, with a well-designed fecal sampling methodology, fecal prevalence is an effective approach to estimate the prevalence of *Echinococcus* species in the Tibetan fox population. With this sampling–analysis protocol, long-term monitoring will allow us to acquire a basic knowledge of the infection status of *Echinococcus* spp. and analyze the prevalence trends for each parasite species in Tibetan fox populations.

While copro-PCR methods are increasingly used for parasite surveillance in wildlife, fecal prevalence without information on individual host identities may lead to misestimating the true population prevalence if a small number of host individuals are sampled repeatedly. This problem can be significant, especially when the wildlife host species has strong territorial and marking behaviors, as do the Tibetan fox [20, 21] and red fox [34]. Therefore, the fecal prevalence of *E. multilocularis* in the coyote was calibrated by identifying the host individuals with an SSR genotyping technique [22]. However, genotyping is costly, especially for large-scale studies with huge numbers of samples tested, because each successfully genotyped sample costs up to US$90–100 in coyotes [22] and about ¥800 (approximately US$112) per fecal sample from the Tibetan foxes in the present study. Moreover, because complex and unpredictable environmental conditions and the presence of inhibitors in feces can reduce the quality of copro-DNA, the success rates of amplification vary significantly among SSR loci. Consequently, only a small proportion (37.6%, 94/250) of Tibetan fox-derived fecal samples were suitable for individual identification (Table 1), and several tests were usually repeated to confirm the genotype at one locus. Furthermore, the optimal SSR loci varied between years, complicating long-term tracking of individuals. Therefore, the high cost and technical challenges make the copro-DNA genotyping technique impractical for large-scale and time-sensitive surveillance programs.

Although individual-based prevalence estimation based on mathematical model predictions and confirmation with empirical data has been advocated [35], a well-designed anonymous fecal prevalence survey remains a more practical approach for empirical studies. The potential disadvantages of anonymous fecal prevalence include: (1) oversampling certain individuals with multiple fecal samples; (2) fecal samples not reflecting the true infection status (e.g., absence of infection) in the same host; and (3) false negative results [22, 36]. To minimize the influence of these disadvantages, scholars must design the sampling scheme carefully according to the spatial behavior characteristics of the studied species in empirical studies [37]. Our key finding is that an optimal transect line interval of 200–900 m gave an unbiased estimate of the prevalence of *Echinococcus* species in the Tibetan fox population (Fig. 2d, Table S3).

However, while this sampling scheme was validated using 2010–2012 data, its application to the 2016–2019 period relies on the assumption that the fox population density remained stable during these two periods. To validate this, our previous study compared the genotyped population size estimates from 2010 to 2012 with the data from line transect surveys (estimated using the DISTANCE model) during the same time period and found no significant difference. Furthermore, our long-term line transect population surveys from 2010 to 2023 have confirmed that the fox population density remained stable (Wang et al., in preparation). The Tibetan fox is a territorial species that usually has a home range size (95% FKE) ≥ 107 ha, equivalent to a circular area with a radius of 584.8 m (Wang et al., in preparation). Therefore, the optimal sampling interval between line transects generated in this study (200–900 m) is appropriate for the local Tibetan fox population in our study area. However, we suggest that the fox population density in the study area must be evaluated to control for any bias in the sampling intervals.

The eastern part of the Tibetan plateau is the *Echinococcus*-endemic region with the highest prevalence of human echinococcosis in the world [18, 38, 39]. Shiqu County is a typical *Echinococcus-*infected area, with a complex transmission system consisting of integrated synanthropic and sylvatic cycles, and epidemiological studies here have mainly focused on the host species and humans involved in synanthropic transmission [40, 41]. However, systematic knowledge of the survival of *Echinococcus* spp. and their coevolution with natural wildlife hosts (in the sylvatic cycle) provides the foundation for strategies to control, prevent, and manage zoonotic disease.

Although surveillance of the prevalence of *Echinococcus* cestodes in wild definitive hosts began as early as the 1990s, epidemiological studies based on technologies that detect fecal prevalence only began in the late 2000s in China. The first record of *E. multilocularis* infection in Tibetan foxes was documented in 1995 from Shiqu County, with a prevalence of 59.1% (13/22) [7]. Based on early studies, a new species, *E. shiquicus*, was discovered in the early 2000s, with a reported prevalence of 37.5% (6/16) in Tibetan foxes [8]. However, these early studies were all based on necropsy, which is inappropriate for large-scale and long-term monitoring, particularly because the Tibetan fox is a nationally protected wildlife species. This led to the adoption of copro-DNA-based fecal analysis for monitoring the population prevalence of *Echinococcus* in Tibetan foxes [10]. In recent years, copro-DNA-based detection methods have been widely used, and the fecal prevalence of *E. multilocularis* and *E. shiquicus* in the Tibetan fox has been reported in various endemic areas in Sichuan and Qinghai provinces [42, 43]. However, these studies only reported the fecal prevalence in local areas for limited periods, rather than long-term systematic monitoring. Moreover, unless the size of the local host population is known, the fecal prevalence of *Echinococcus* may deviate significantly from the true population prevalence. Indeed, using the same fecal samples, an overall fecal prevalence of 62.0% was reported in 2010 from Shiqu County [10], which was 12% higher than the genotyped population prevalence (Table 2). Therefore, they noted that without calibration to the true host population prevalence, bias in fecal sampling and consequently in fecal prevalence is inevitable [10]. Nevertheless, although the nested PCR protocol employed in this study provides a reasonable solution, it suffers from the disadvantages of the lower sensitivity of the traditional PCR (Table 1). In recent years, the utilization of real-time PCR has become increasingly prevalent, and offers significant advantages in sensitivity and quantitative analysis, as demonstrated for *E. multilocularis* [47]. The development and validation of such an assay represent a key priority for enhancing future molecular surveillance of *Echinococcus* dynamics on the Tibetan Plateau.

Our data since 2010 provide basic information on the infection burden of the two *Echinococcus* species and their long-term trends in the Tibetan fox population. We detected a weak but significant decreasing trend in the prevalence of *E*. *multilocularis* (Table 2). Similar trends were also detected in the intermediate host species (e.g., lagomorphs and rodents) [44] and domestic dogs in the same area [12]. The Chinese central government has started a pilot echinococcosis prevention and control project in Shiqu County since November 2015 [45]. Could the decreasing prevalence trends in animal host species, especially after 2015, be related to the carrying out of the project, and how will this project finally influence the cycling of echinococcosis in the wildlife reservoir? Our data indicate a significant discrepancy between the prevalence of *E. multilocularis* and *E. shiquicus*. The increasing prevalence of *E. shiquicus* and the decreasing prevalence of *E. multilocularis* were not just detected in Tibetan foxes (Table 2), but have also been confirmed in the intermediate host community (e.g., lagomorphs and rodents) in the same area and period [44]. Two *Echinococcus* species present in the same individual host may cause significant interspecific competition [46]. What then is the long-term interaction pattern between these two *Echinococcus* species, and what is the mechanism of their coexistence? More importantly, the transmission of both parasite species is intrinsically linked to the population dynamics of small mammal intermediate hosts [48], and may be significantly influenced by anthropogenic disturbance [49–51]. To solve these issues, integrative approaches consistent with the One Health concept are necessary to develop and implement. As a fundamental contribution, long-term monitoring of the prevalence in populations of host species will provide effective data and early warning information.

## Conclusions

Optimized line transect fecal sampling offers a robust and efficient method for estimating the prevalence of *Echinococcus* spp. in their definitive hosts, particularly in remote, protected wildlife populations. This methodology provides a practical framework for the long-term and large-scale monitoring of parasite prevalence in these communities, facilitating the development of effective surveillance and management strategies. Notably, the optimized sampling interval developed in this study is predicated on the stability of the host populations. If the host population density is unstable between sampling years, the optimized sampling interval may need to be calibrated based on host individuals detected using copro-DNA SSR techniques. Moreover, this study also demonstrated a persistent, albeit declining, high prevalence of *E. multilocularis* in the Tibetan fox population in Shiqu County from 2010 to 2019, which indicates a continuing, significant risk of sylvatic alveolar echinococcosis. Further research is required to clarify the interspecific competition and distinct transmission dynamics of *E. multilocularis* and *E. shiquicus*, and particularly their potential for zoonotic spillover and the implications for public health.

## Supplementary Information

Additional file 1.

Additional file 2.

## Data Availability

All data generated or analyzed during this study are included in this published article and supplementary materials.

## Abbreviations

BamHI: *Bacillus amyloliquefaciens* H strain I
BSA: Bovine serum albumin
*cox1*: Cytochrome c oxidase subunit 1
CI: Confidence interval
*CYTB*: Cytochrome b
DNA: Deoxyribonucleic acid
ddH_2_O: Double-distilled water
*E. multilocularis*: *Echinococcus multilocularis*
*E. shiquicus*: *Echinococcus shiquicus*
*E. granulosus*: *Echinococcus granulosus*
FKE: Fixed kernel estimation
GPS: Global positioning system
HaeIII: *Haemophilus aegyptius *III
Sry: Sex-determining region Y
SSR: Simple sequence repeat
PIC: Polymorphism information content value
He: Expected heterozygosity
Ho: Observed heterozygosity
PCR: Polymerase chain reaction
P_ID_: Probability of identity
P_IDbiased_: The probability of identity for individuals randomly mating
P_IDunbiased_: The probability of identity with sample size corrected
P_IDsibs_: The probability of identity for related individuals
RFLP: Restriction fragment length polymorphism
SspI: *Sphaerotilus* species I
ZFX: X-linked zinc finger protein
