# Supplementary material for: Estimating the prevalence of Echinococcus spp. in a Tibetan fox (Vulpes ferrilata) population on the eastern Tibetan Plateau
Source: Parasit Vectors. 2026 Jan 21;19:41. doi: 10.1186/s13071-025-07085-3 (PMC12822117; doi:10.1186/s13071-025-07085-3)
Supplement: Supplementary file 1 — Additional file 1. [file 13071_2025_7085_MOESM1_ESM.pdf]

(a)

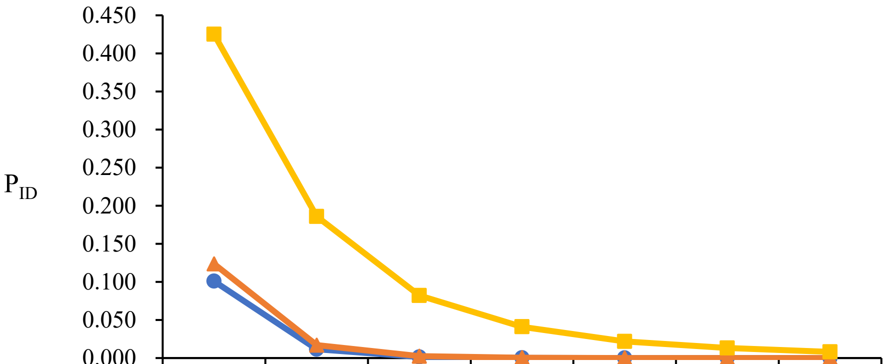

	p01i	p02d	CXX172	p08	p05h	Gender	AHT-142
PIDunbiased	0.101	0.011	0.001	0.000	0.000	0.000	0.000
PIDbiased	0.124	0.017	0.002	0.000	0.000	0.000	0.000
PIDsibs	0.425	0.186	0.082	0.041	0.022	0.013	0.008

Multiloci P_{ID} in distinguishing Tibetan fox feces in 2010

(b)

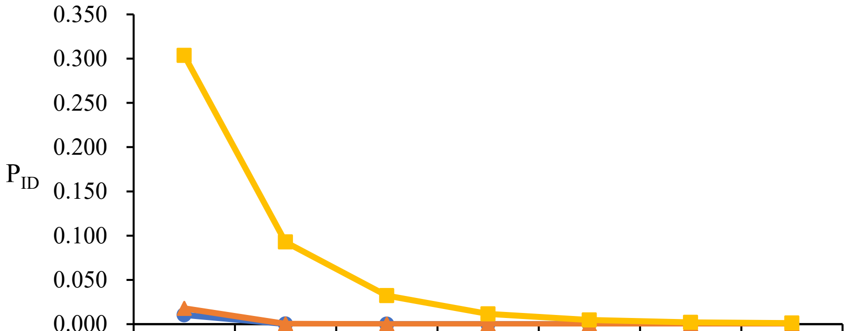

	CXX172	P03	P01i	CPH6	P08	CPH8	Gender
PIDunbiased	0.010	0.000	0.000	0.000	0.000	0.000	0.000
PIDbiased	0.018	0.000	0.000	0.000	0.000	0.000	0.000
PIDsibs	0.304	0.093	0.032	0.012	0.005	0.002	0.001

Multiloci P_{ID} in distinguishing Tibetan fox feces in 2011

(c)

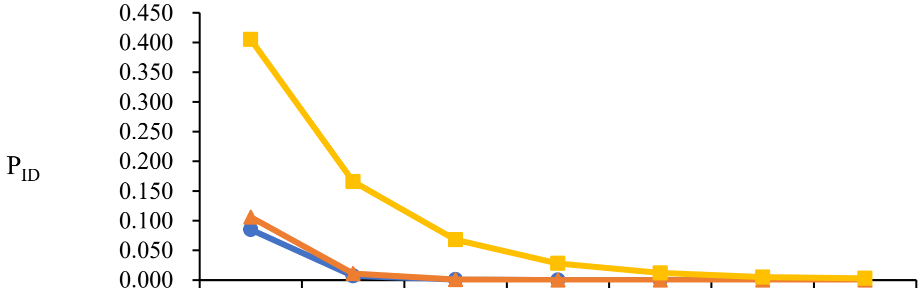

	CPH08	CPH6	P03	P08	CXX17 2	P01I	Gender
PIDunbiased	0.085	0.007	0.001	0.000	0.000	0.000	0.000
PIDbiased	0.106	0.011	0.001	0.000	0.000	0.000	0.000
PIDsibs	0.405	0.166	0.068	0.028	0.012	0.005	0.003

Multiloci P_{ID} in distinguishing Tibetan fox feces in 2012

Fig. S1 Probability of identity (P_{ID}) values for the combined loci used to identify individual Tibetan foxes from fecal samples collected in 2010 (a), 2011 (b), and 2012 (c)

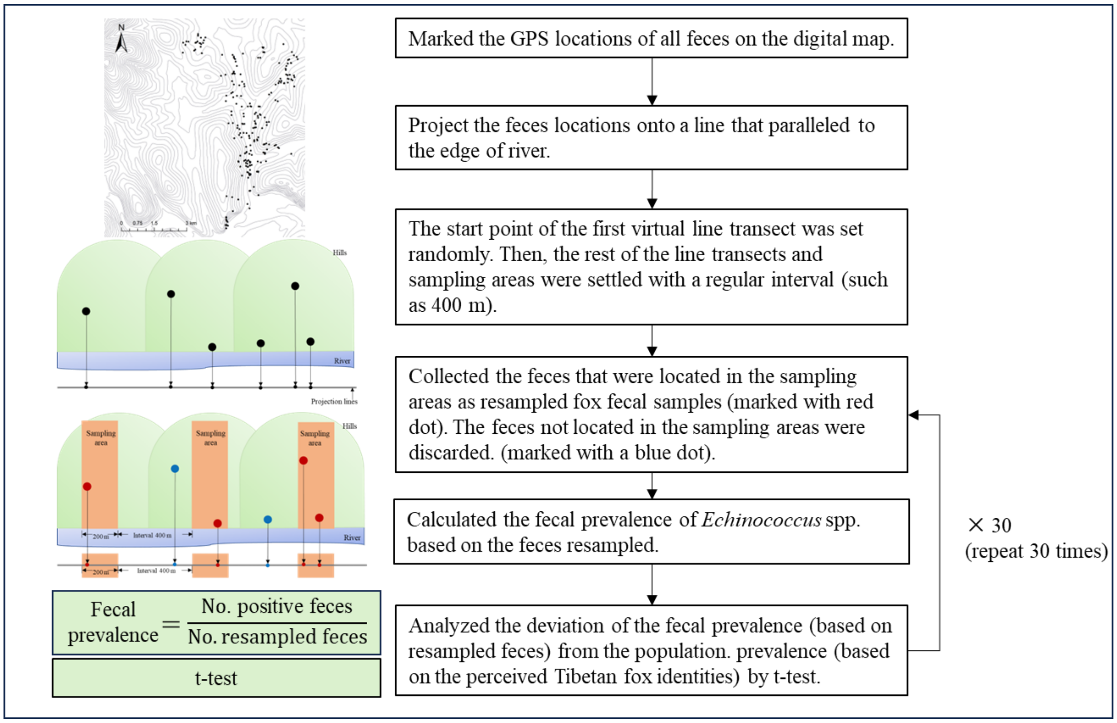

Fig. S2 Protocol for estimating the range of optimal intervals between line transects when sampling feces

Table S1 Characterization of genetic markers used in fox identification and *Echinococcus* spp. detection

	Species	Gene/Locus	Primers	Ta (℃)	size (bp)	Reference
Species identification	Tibetan fox	Cyt *b*	GATATGAAAAACCATCGTTG	55	478	[1]
			AAACTGCAGCCCCTCAGAATGATATTTGTCCTCA			
Sex identification	Tibetan fox	ZFP gene	CAAAAGGTGGCGATTCAATAA	58	195	[2]
			ATGGAGAGCCACAAGCTGAC			
		Sry	CTCGCGATCAAAGGCGCAAGAT	58	103	[3]
			TTCGGCTTCTGTAAGCATTTTC			
Microsatellite genotype	Tibetan fox	AHT-142	AAGCAGATCCTAGAGCAGCA	58	133-148	[4]
			CCCCACAGTTTAGAAATATCTGC			
		Cph1	GCCTAGCCCAGTGAAAGTTAC	52	135-144	[5]
			TTCCAATGCCTGATAACTGAGA			
		Cph6	CATTGGCTGTTTGACTCTAGG	58	107-136	[5]
			ACTGATGTGGGTGTCTCTGC			
		Cph8	AGGCTCACAATCCCTCTCATA	58	186-200	[5]
			TAGATTTGATACCTCCCTGAGTCC			
		Cxx172	CCTGTCTCCTGTGGACCAAT	58	155	[6]
			ACATGCAAAAGGACACATTACG			
		DB1	CCCAATACAGCAAGACCTCT	58	141-159	[7]
			ACCTACTCTGCACAGAGAAG			
		P01f	GCCTGGGAAGGAATCAAG	64	170-230	[7]
			CCTGGCTCCATCCAAACA			
		P01i	GGGGACCTCAAGAATGT	58	164-232	[8]
			TGTCTCATCAATGCCAAG			
		P02	GTGACGCCCAAACATCC	58	140-168	[7]
			GGCTCCTGCTCCTCTGC			

		P02c	AGAAGCAGGATTCACACC<br>TTCCCTCAACACTCACC	54	180-222	[7]
		P02d	CTTATTGGGCTTCTTGG<br>TTGCCTCAGCATCTCATC	60	124-144	[7]
		P02h	CTGGGAAGGAAGCAAGA<br>CATACACCCTAAGCAACTG	58	195-207	[7]
		P03	GAAAGCCAGGGTCCAGTAG<br>CAGAAACGGGCATCCATA	58	192-208	[7]
		P03h	TCTACCCATACAGCATCC<br>GAGCCAGTGTCTATCCCT	58	226-236	[7]
		P03i	ATCTCCTCCAAGACCTCC<br>TCCCACCCCTGATACCT	58	447-163	[7]
		P04c	GATCAAGTCCCAGGTCG<br>CACTGAGTTAGCCAGATGC	56	170-280	[7]
		P04h	CTGCTGGAAAGAGGAAT<br>CAGAGCCAAAGGTAGGT	58	203-235	[7]
		P05h	TCACTCCTAAGAAATCGGG<br>GCACTGGCATGAACTGG	58	310-336	[7]
		P06h	GGATGGGCAGATGGAGCA<br>GCCTGAGCAGTTGACCTTG	58	281-313	[7]
		P08	ATTCTGGACCAATGAGGC<br>GGAGGGGAGGAAGGATA	62	170-210	[7]
		P09	GGTGGAGACTGCTTGGGAT<br>CCTGTGGAATAGAGCGGGT	58	300-336	[7]
Echinococcus detection	*E. granulosus*	EgG1 Hae III (external)	ACACCACGCATGAGGATTAC<br>ACCGAGCATTTGAAATGTTGC	55	269	[9]

	EgG1 Hae III (internal)	GAATGCAAGCAGCAGATG<br>GAGATGAGTGAGAAGGAGTG	55	133	[9]
Taeniidae	Taeniidae	TTGAATTTGCCACGTTTGAATGC<br>GAACCTAACGACATAACATAATGA	52	874	[10]
E. multilocularis	*cox1*	GTCATATTTGTTTAAGTATAAGTGG<br>CACTCTTATTTACACTAGAATTAAG	52	243	[11]
E. shiquicus	*cox1*	GTTGGTTACGTTACCGGTT<br>TCTTATTAACATTTGAATTCAAC	52	420	[12]

Reference

1. Wayne RK, Geffen E, Girman DJ, Koepfli KP, Lau LM, Marshall CR. Molecular systematics of the canidae. Mol Sys Biol.1997;46:622-653. https://doi.org/10.1093/sysbio/46.4.622.
2. Ortega J, Franco MR, Adams BA, Ralls K, Maldonado JE. A reliable, noninvasive method for sex determination in the endangered San Joaquin kit fox (*Vulpes macrotis mutica*) and other canids. Conserv Genet.2004;5:715-718. https://doi.org/10.1007/s10592-003-1862-5.
3. Jiang WB, Liu N, Zhan GT, Renqing PC, Xie F, Li TY, et al. Specific detection of *Echinococcus* spp. from the Tibetan fox (*Vulpes ferrilata*) and the red fox (*V. vulpes*) using copro-DNA PCR analysis. Parasitol Res. 2012;111:1531-1539. https://doi.org/10.1007/s00436-012-2993-8.
4. Wandeler P, Funk SM. Short microsatellite DNA markers for the red fox (*Vulpes vulpes*). Mol Ecol Notes. 2006;6:98-100. https://doi.org/10.1111/j.1471-8286.2005.01152.x.
5. Fredholm M, Wintero AK. Variation of short tandem repeats within and between species belonging to the Canidae family. Mamm Genome. 1995;6:11-18. https://doi.org/10.1007/BF00350887.
6. Ostrander EA, Sprague GF, Rine J. Identification and characterization of dinucleotide repeat $(CA)_n$ markers for genetic mapping in dog. Genomics. 1993;16:207-213. https://doi.org/10. 1006/geno.1993.1160.
7. Holmes NG, Mellersh CS, Humphreys SJ, Binns MM, Soliman AH, Curtis R, et al. Isolation and characterization of microsatellites from the canine genome. Anim Genet. 1993;24:289-292. https://doi.org/10.1111/j.1365-2052.1993.tb00313.x.
8. Li M, Wang XM, Jiang WB, Hua PY, Wang ZH. Isolation and characterization of fifteen microsatellite loci in the Tibetan fox (*Vulpes ferrilata*). J Genet. 2011;90:82-85. http://www .ias.ac. in /jgenet/OnlineResources/90/e80.pdf.
9. Abbasi I, Branzenburg A, Campos-Ponce M, Abdel HSK, Raoul F, Craig PS, et al. Copro-diagnosis of *Echinococcus granulosus* infection in dogs by amplification of a newly identified repeated DNA sequence. Am J Trop Med Hyg. 2003;69:324-330. https://doi.org/10.4269/ajtmh.2003.69.324.
10. Nakao M, Sako Y, Yokoyama N, Fukunaga M, Ito A. Mitochondrial genetic code in cestodes. Mol Biochem Parasitol. 2000;111:415-424. https://doi.org/10.1016/s0166-6851(00)00334-0.

11. Nonaka N, Hirokawa H, Inoue T, Nakao R, Ganzorig S, Kobayashi F, et al. The first instance of a cat excreting *Echinococcus multilocularis* eggs in Japan. Parasitol Int. 2010;57: 519-520. https://doi.org/10.1016/j.parint.2008.07.001.
12. Nakao M, McManus DP, Schantz PM, Craig PS, Ito A. A molecular phylogeny of the genus *Echinococcus* inferred from complete mitochondrial genomes. Parasitology 2007;134: 713-72. https://doi.org/10.1017/S0031182006001934.
